# The double-edged sword of abortion regulations: Decreasing training opportunities while increasing knowledge requirements

**DOI:** 10.1080/10872981.2022.2145104

**Published:** 2022-11-14

**Authors:** Hillary J. Gyuras, Meredith P. Field, Olivia Thornton, Danielle Bessett, Michelle L. McGowan

**Affiliations:** a Gyuras Is a Research Associate, College of Public Health, the Ohio State University, Ohio, USA; b Division of Social Sciences, Alfred University, Alfred, New York, USA; c Department of Obstetrics and Gynecology, Brigham and Women’s Hospital, Massachusetts, USA; d Department of Sociology, University of Cincinnati, Cincinnati, Ohio, USA; e Biomedical Ethics Research Program, Department of Quantitative Health Sciences, Mayo Clinic, Rochester, Minnesota, USA

**Keywords:** Abortion, abortion restrictions, medical education, residency, medical school, fellowship, training, qualitative research

## Abstract

**Purpose:**

The authors explore how abortion regulations in Ohio, an abortion-restrictive state in the USA, impact obstetrician-gynecologists’ (OB/GYNs) training in reproductive healthcare and describe what OB/GYNs believe to be the broader impact of Ohio’s regulations on skill-building, skills maintenance, and professional retention of reproductive healthcare providers in the state. Authors discuss how their findings foreshadow abortion training limitations in Ohio and other abortion-restrictive states now that abortion regulations have returned to the states.

**Methods:**

The authors conducted four semi-structured focus groups and five in-depth interviews between April 2019 and March 2020. Participants included OB/GYNs practicing obstetrics and gynecology in Ohio between 2010 and 2020. Thematic analysis was conducted using Atlas.ti.

**Results:**

Twenty attending physicians and 15 fellows and residents participated in the study. Participants discussed the impact of Ohio’s written transfer agreement, gestational-limit, and abortion method and facility bans on training and skill-building opportunities. Participants felt that Ohio’s strict abortion regulations 1) limit opportunities to observe and perform abortion procedures during training; 2) require learning the ever-changing legality of abortion provision; 3) limit the number of abortions OB/GYNs can provide, leading to the atrophy of their skills over time; and 4) may prevent prospective medical students and residents from choosing to study in Ohio and may lead to physician attrition from the state.

**Conclusion:**

Prior to the reversal of federal protections for abortion in 2022, OB/GYNs in Ohio and other abortion-hostile states experienced barriers to training in abortion care. In returning abortion regulation to the states, access to training is likely to be increasingly restricted. This research demonstrates how abortion-restrictions hamper physicians’ skills needed to care for patients, particularly in emergent situations. This puts patients at risk and places physicians in precarious ethical positions. Expanding protections and reducing restrictions on abortion will ensure OB/GYNs and trainees have the skills necessary to care for patients presenting for reproductive healthcare.

## Introduction

On 24 June 2022, the Supreme Court of the USA (U.S). upended decades of legal precedent when it overturned *Roe v Wade* and *Planned Parenthood v Casey*. The Justices’ decision in *Dobbs v Jackson Women’s Health Organization* returned the right to regulate abortion back to individual states[1]. Although abortion restrictions have proliferated in the U.S. since 2011, 26 states were expected to completely outlaw abortion after the decision[2]. Many of these states have indeed taken steps to outlaw or severely restrict abortion; while some have been successful, others have been met with legal challenges [3,4].

Prior to the decision, experts anticipated that access to abortion training for obstetricians and gynecologists (OB/GYNs) would diminish significantly if *Roe* and *Casey* were overturned [5–8]. While the Association for Professors of Gynecology and Obstetrics (APGO) *recommends* including pregnancy termination in medical school curricula and the Accreditation Council for Graduate Medical Education (ACGME) *requires* that OB/GYN residents have access to training in abortion provision, such training in the U.S. has historically been variable – with a plethora of studies highlighting differences in training accessibility based on one’s region of work or study and institutional religious affiliation [9,10].

For example, studies published in 2011 showed that medical students, especially those attending religiously-affiliated schools, reported dissatisfaction with the training they received on family planning topics including abortion, sterilization, and contraception [11,12]. As such, many medical students have relied upon family planning electives to learn about these topics[13].

A 2008–2009 survey of residents in several midwestern states found that those in faith-based programs were less likely to be satisfied with their family planning training than their peers in non-faith-based programs[14].

In addition, residents in the southern region of the U.S. had less training on dilation and evacuation (D&E) procedures, the most common second-trimester abortion method, than residents in other regions of the U.S., especially those in northeastern states[15]. In more recent studies, nearly half of residency program directors in Catholic schools reported access to abortion training was ‘poor’ when surveyed; more than one-quarter additionally reported that their programs did not meet specific ACGME requirements[16].

Despite these findings, research shows improved access to abortion training for residents – even in the South and Midwest – since the Kenneth J. Ryan Residency Training Program was established in 1999 [17–19]. Nearly 7,000 OB/GYN residents have trained with the program that ensures they have access to family planning rotations, and Ryan residency program directors have reported that their residents were competent in abortion and contraceptive care and that their programs were more desirable to applicants[19]. Importantly, residents with access to abortion training reported feeling more prepared to manage pregnancy loss and complications[20]. Still, additional surveys of residency program directors indicate hospital policies (both informal and formal), state laws, and a lack of faculty able to provide training continue to create barriers to abortion education [21,22].

Regardless of constraints on training, skills applicable in abortion provision are widely used across obstetric and gynecological practice. They are utilized for management of miscarriage, ectopic pregnancies, fetal demise, and other cases wherein a pregnancy may harm the health of a pregnant patient. Moreover, abortion is common, and hundreds of thousands of people seek abortion care per year. For example, Guttmacher reports there were 930,160 abortions in the U.S. in 2020 – up eight percent from 2017[23].

In this paper, we examine how OB/GYNs in one abortion hostile state, Ohio, experienced abortion restrictions in the years prior to the *Dobbs* decision. While we have previously reported on how state abortion laws hinder physicians from exercising clinical judgment and cause ethical dilemmas[24], here we consider what broader consequences these regulations and subsequently limited training opportunities have had on physicians. The two main objectives of this paper are to describe 1.) how Ohio’s abortion regulations affect OB/GYNs’ training in reproductive healthcare; and 2.) what OB/GYNs believe to be the impact of Ohio’s regulations on skills building, skills maintenance, and professional retention of OB/GYNs in the state. We then explore the expected impact of these regulations and training limitations in Ohio and other abortion-hostile states in the post-*Dobbs* era.

## Methods

We conducted semi-structured focus groups and interviews with OB/GYNs from across Ohio. We recruited study participants with at least six months of experience practicing obstetrics and gynecology in Ohio between 2010 and 2020, from hospitals affiliated with Ohio universities, medical specialty professional societies, and advocacy groups via direct email and snowball sampling. Because most research that involves abortion providers is focused on physicians at abortion clinics, we were interested in the experiences of OB/GYNs working at abortion clinic-adjacent facilities. Consequently, OB/GYNs who worked in free-standing abortion clinics at the time of the study, including Planned Parenthood clinics, were ineligible to participate.

We conducted four semi-structured focus groups with a total of 30 participants and in-depth interviews with five participants between April 2019 and March 2020. Focus group discussions lasted 90 minutes. Participants who were unable to attend scheduled focus groups were offered individual interviews, each of which lasted 45–60 minutes. Focus groups and interviews utilized the same moderator guide (Appendix 1).

Participants included 20 attending physicians and 15 fellows and residents who were based in Ohio’s most populous regions. While a small number practiced privately, most of the 35 study participants worked in public, not-for-profit community hospitals, and academic medical centers. Most participants volunteered that they had current or previous experience providing abortion care in hospital settings, and some had previously worked in abortion clinics.

Because little is known about the extent to which abortion-clinic adjacent healthcare professionals experience abortion regulations, and to generate conversation about the specific pieces of legislation that impacted their work, the study moderator gave participants a legislative timeline describing state abortion laws that were enacted, enjoined, or proposed in Ohio between 2011 and 2019[25]. After acquiring verbal consent to participate in the study, we asked participants to review the timeline and discuss which pieces of legislation had impact on their professional practice. We audio-recorded each focus group and interview and transcribed the recordings. Each transcript was deidentified and participants were assigned pseudonyms. Two members of the research team (HG and MF) conducted a thematic analysis of transcripts using ATLAS.ti, and then met to review themes and reach consensus[26]. We collected data until we reached theoretical saturation.

## Results

Participants discussed a range of Ohio’s laws but focused on three pieces of enacted and two pieces of enjoined legislation that had or could impact training and skill-building opportunities (Table 1). Our participants discussed four key ways that Ohio’s abortion regulations and institutional interpretations of these regulations affect medical students and OB/GYN trainees. First, Ohio’s abortion regulations limit the availability of opportunities for trainees to observe and perform abortion procedures. Second, regulations require trainees to track the ever-changing legality of abortion provision. Third, participants felt Ohio’s abortion regulations led to the atrophy of their skills over time as they were, by law, severely limited in their ability to provide abortion care within their institutions. Finally, participants agreed that the state’s strict regulations would discourage medical students and residents from choosing to study in the state and may also prompt physicians already practicing in Ohio to leave in order to practice in less restrictive environments.

**Table 1.** Ohio laws that impact access to abortion training.

Regulation	Description	Year
§ 5101.57	**Public Facilities Ban**: Bans public facilities from providing ‘nontherapeutic’ abortions. **Enacted.**	2011
§ 3702.3010	**Transfer Agreement**: Requires abortion clinics to secure written transfer agreements with hospitals within 30 miles of the clinic. **Enacted.**	2015
§ 2919.201	**20-week ban**: Bans abortion after 20 weeks post-fertilization or 22 weeks since a pregnant person’s last menstrual period; includes exceptions for medical emergencies. **Enacted.**	2017
§ 2919.15	**D&E Ban**: Bans dilation and evacuation procedures. Partially enjoined.*	2018
§ 2919.195	**6-week ban**: Prohibits abortions after detectable heartbeat. Enjoined.**	2019

*Partially enjoined at the time of the study. This went into effect 24 June 2022. It remains in effect as of this writing. **Enjoined at the time of the study, this went into effect 24 June 2022. It was temporarily enjoined again on 14 September 2022 and remains enjoined as of this writing.

### Ohio abortion regulations limit training opportunities

Throughout our study, participants emphasized the importance of being trained to provide abortion care as a component of comprehensive reproductive healthcare. Dylan, a resident in central Ohio, said:

*I knew that it was an important part of training, not necessarily because I wanted to focus my practice on that afterwards, but we’ve seen cases where this is a lifesaving procedure. And if we don’t know how to do that, we’re not prepared to go out and be that sole provider. So, I think it’s a very important, necessary thing.*

Dylan’s comment is illustrative of how participants valued access to abortion training and viewed abortion provision as a skill necessary for comprehensive patient care.

Ohio’s OB/GYN trainees must decide whether to accept the limited exposure they will receive in their programs or supplement it through extra clinical rotations or post-residency fellowships. Some participants discussed seeking extra clinical rotations outside of their programs. A resident in central Ohio, Carol, discussed her previous experience as a medical student in Ohio:

*I, as a third-year med student, was able to rotate at Planned Parenthood. It was like an optional rotation, and I elected to go, and so I got … better information than certain people, because I went there and talked with the providers who actually are providing the services. … I think you kind of had to seek that information out though. It wasn’t something that in medical school, you really were taught.*

Another participant, Carla, said that even though her residency program in northeastern Ohio included a family planning rotation, there were too few abortions performed at her home institution to provide adequate training. She explained:

*Luckily, we do have … a family planning rotation … we get to [go to] Planned Parenthood and [an independent abortion clinic], but it’s still not enough … . We go to [independent abortion clinic] once a week and Planned Parenthood once a week, but not having it at our primary institution, involved in all of our rotations, which is what I think it should be … I don’t think we get enough of that training. A lot of the residents who want to go into family planning will do outside electives to get that training that they feel like they need.*

These quotes demonstrate how seeking additional training opportunities may be beneficial but inadequate to fully prepare trainees for patient care.

Tamara, a resident in northwestern Ohio, illustrates how trainees experience different challenges accessing abortion training – in some cases, precluding training on certain types of abortion entirely – based on the region in which they train. Tamara’s residency program partners with an independent abortion clinic that ceased providing procedural abortions after it was unable to comply with the state’s written transfer agreement law (§ 3702.3010)[27]. While she was happy that she had the opportunity to rotate at the clinic, she felt disappointed that she would not get experience with procedural abortions. She explained, *‘ … it was better when … they did surgical abortions. … it’ll be good to see [medication abortions], but I’m not really going to learn that much that I don’t know.’* Thus, limited clinic operations and exposure to a small number of procedural abortions within her residency program at a public institution barred from providing nontherapeutic abortion care conspire to further limit Tamara’s training on a broad spectrum of abortion care. Other medical students and residents in the region who rely upon this clinic for their abortion education will similarly receive limited training.

Participants repeatedly discussed Ohio’s public facilities ban (§ 5101.57) and dilation and evacuation (D&E) ban (§ 2919.15) as regulations that have significantly reduced training opportunities. In some cases, participants observed a small number of D&Es during residency, and in other cases they used their limited elective time to get additional training on D&Es outside of their primary institution. Tracy, who completed residency in northeast Ohio, said, *‘ … to get trained on D&E procedures, you actually had to do a separate elective rotation which was your only elective time during residency. You had to sort of set aside your only elective block for the entire four years of residency in order to get trained on that procedure.’*

Ohio abortion regulations burdened trainees with finding workarounds to be trained adequately in D&E, which they viewed as a life-saving procedure that all OB/GYNs should learn to perform. Mary, a resident in southwestern Ohio, explained:

*D&E is a life-saving procedure, and I only got to see two during my residency, and … I fought to see those two. … you know that at some point, something could happen where even if the baby is already demised, the patient is hemorrhaging, or some other scenario, that this would be the safest and most reasonable lifesaving technique for the patient.*

Mary highlights how, in banning public facilities from performing ‘nontherapeutic’ abortions, policymakers are risking the lives of patients, because many OB/GYNs cannot get adequate training, and consequently, cannot perform D&Es in emergency situations. Several participants stated that only one or two attendings in their institutions were skilled enough to perform D&Es in any situation.

### Ohio abortion regulations require learning the ever-changing legality of abortion provision

Another theme that emerged was that Ohio abortion regulations increase the amount of regulatory knowledge that OB/GYNs should have when they complete their training. Every new abortion regulation, and any change to the legal status of an existing one, represents additional information that OB/GYNs must learn. Furthermore, they must understand both how their institution interprets the regulation and what policy the institution implements (or not) as a result.

The majority of participants agreed that their training programs educated them about neither abortion regulations nor institutional interpretations of regulations. Consequently, trainees must identify alternate sources of clear and unbiased information about regulations. Individual trainees accomplish this to varying degrees, leaving some worried about the possibility of unknowingly breaking the law. Discussing their experience as a medical student, Anna, who completed both medical school and a fellowship in Ohio, said:

*And it’s hard as a medical student, ‘cause you don’t always know exactly what the rules are. You’re just kind of going based on what people tell you is what the case is, and at the time I remember being told that … the hospital didn’t do abortions and … I was told they didn’t do them because they didn’t want to take on that … type of like legal responsibility was how it was sort of conveyed to me.*

In this case, Anna’s former institution interpreted state law conservatively, as the law would have permitted abortions in some instances, and she was not given any information about the specifics of actual regulations.

Participants commonly agreed that their programs should include education about interpreting medical laws given Ohio’s complex regulatory landscape. To the agreement of other participants, Deb, a resident in central Ohio, shared that, *‘in medicine, you’re trained … how to learn medicine, or what resources you should use if you need to study this or learn that … But … I, at least, didn’t necessarily have as much training in “Here’s how to interpret … medical law” … So, I think this is maybe an area that’s … lacking in medical education in general.’*

Without their programs providing education in medical law, participants sought other sources of information, often news outlets or public advocacy groups. Carla, a resident in northern Ohio, relayed sources of information upon which they relied: *‘It’s hard in residency. I try to watch the news as much as I can. A lot of my friends are … also involved in that kind of stuff … it does come up on social media, so I do get a lot from there too … ’* Some residents noted that they relied upon their attendings’ understanding of abortion laws. Unfortunately, participants who were attendings also expressed confusion and struggled to keep up with the ever-changing laws. Thus, both residents and attendings felt that they have difficulty accessing concise, applicable information regarding interpretation of abortion law.

Sandy, a resident, emphasized how not having continuous, reliable information puts both patients and physicians at risk:

*Right after the D&E ban was officially passed and right around the time they [the courts] were figuring out injunctions and stuff, we had a patient come in for … a miscarriage, which regardless of the method would have been okay at that point. But it was around 14 weeks. We were going to do a D&C and ended up doing kind of like a modified D&E, and right before we started doing that part, my chief resident turns to me and goes, ‘Wait we’re allowed to do this right?’ Because … in terms of that information, it’s just not widely available or widespread.*

The absence of widespread reliable information left individual OB/GYNs of all levels of experience feeling responsible for deciphering healthcare policy, unsure that what was clinically indicated was legally permissible, and vulnerable to unknowingly breaking the law.

### Ohio abortion regulations lead to the atrophy of clinical skills over time

Participants also revealed that their procedural abortion skills atrophied over time because state law prevented them from performing a sufficient number of abortions to maintain them after residency. Pamela, an attending physician in a public institution in northeast Ohio, said:

*It’s frustrating because we have lots of faculty here that are trained on doing this. We learned it in residency … and then the only [abortions] we are allowed to do are the ‘medically indicated ones’ … and at some point, you aren’t skilled if you’re not doing enough of them. … there are a few of us that will do second trimester D&Es, and every time we have to have this internal conversation of ‘are we the right person for this patient or should we send her elsewhere, even in this awful, traumatic time where she just got diagnosed with a demise, because we’ve only done one or two a piece, each, in the past year. … are we still the most skilled surgeons?’ And I think that’s frustrating that we are losing clinical skills that are really necessary for health care.*

The lack of case volume and access to training reduced the number of physicians qualified to perform D&Es within the participants’ institutions of employment. In one focus group, Crystal, a resident, expressed concern that only one attending at her hospital is trained in providing D&Es. In response, Tammy, an attending, shared that she *is* the person within her institution who is called upon to perform D&Es in emergent situations. She said, *‘it gets scary because … if I’m the only person, if I leave, who is going to provide that [type of abortion]? If I can’t be available? If I need help … ?’*

Consequently, finding a skilled physician to perform a procedure delays patient care in emergent situations, which participants reported puts their patients at risk. For example, Erin, an attending in southwest Ohio, noted, *‘when we have someone that is hemorrhaging, and she needs a D&E, it’s like we have to find the two or three people that know how to do it, and if they’re out of town or they’re not there … we’re in trouble.’* Tammy and Erin’s stories elucidate both the stress placed upon the few physicians capable of providing D&Es in emergent situations as well as the risk to patients’ health.

### Ohio abortion regulations may prevent trainees from studying in Ohio and lead to physician attrition from the state

In all, participants reported that Ohio’s abortion restrictions could lead to an exodus of OB/GYNs who provide abortions and that some trainees who recognize that regulations limit access to abortion training would avoid applying to medical schools or residency programs in states with more restrictions. Heather, a fellow in northeastern Ohio, recalled:

*I was at the [medical school’s] medical student resident fellow meeting, and the medical students were talking about it. People who were planning to go into OB, and saying they’re not ranking [university in Ohio], Ohio schools or Ohio programs, or ranking them really low, because they wouldn’t get adequately trained in the full spectrum of OB/GYN care.*

Heather’s quote highlights the perceived role of abortion restrictions in deterring future OB/GYNs from training and working in Ohio.

Similarly, Tamara, a resident in northwest Ohio said, *‘ … I’m glad I’m training in Ohio, but … most of me wants to leave Ohio if it keeps going like this … I hate that … everyone who would provide good care to these patients is going to leave because they’re annoyed about the laws.’* Participants stated that they do not want to work in a state where their professional community is under attack and comprehensive reproductive healthcare practice is constrained.

Anna attended medical school in Ohio but completed residency outside of the state. In our interview, she estimated that she performed hundreds of abortions during residency. When she returned to Ohio for fellowship, she was prohibited from providing abortion care. She recalled:

*And as a fellow … I actually had this weird feeling of having come from the residency I came from and having done so many abortion procedures that … I was like, ‘wow, I’m probably one of the best trained abortion providers in this state right now, and I’m not doing any abortions.’ And I couldn’t find a space to carve that into my … schedule as a fellow, and I always felt kind of guilty that that was … a skill I wasn’t using, and I wasn’t able to … provide [abortion] care …*

Anna was not able to use her expertise to provide this care for patients in Ohio nor share it with other physicians who lacked similar training. Ultimately, she left to practice in another state after fellowship – taking her skills and expertise along with her.

## Discussion

Our findings echo previous research on the availability of abortion education in medical schools and residency programs [11–15]. Our participants similarly felt that their programs did not provide adequate information on abortion or abortion laws, that expanding their knowledge base and skillset pertaining to abortion necessitated electives or supplemental trainings, and that they were inadequately trained to provide D&Es [11–15]. In addition, our participants drew clear connections between the deficiencies in their training and Ohio’s abortion regulations.

Participants felt that Ohio’s strict regulations limited the number and types of procedures they were able to observe and perform because the law limits abortions in public academic settings. While some participants sought out electives in order to learn more about abortion care, they felt this process was inefficient and burdensome. Our participants also noted that even after receiving adequate training, abortion restrictions led to an atrophy of their skills and left their patients underserved by limiting the number of OB/GYNs able to provide care in emergent situations. Finally, our participants expressed concern, and provided anecdotal evidence, that some prospective medical students and residents would choose not to train in Ohio, because the hostile regulatory environment would prevent them from receiving comprehensive training in abortion care. At the same time, they anticipated that some individuals who did train in Ohio would ultimately leave to practice in other states where they would not be as restricted by abortion laws.

Our data suggest the state should create a more hospitable regulatory climate for physicians: one that does not include laws that are restrictive, confusing, and threaten physicians with legal penalties for providing abortions. Yet, in the wake of the *Dobbs* decision, Ohio successfully banned abortions after the presence of embryonic cardiac activity on ultrasound for nearly three months and is working to implement further restrictions [3,4]. Our data foreshadow that this will burden Ohio’s trainees by demanding they keep up with ever-changing regulations and further curtailing their training opportunities. OB/GYNs and OB/GYN trainees in similarly restrictive states will face comparable challenges.

While all OB/GYNs seeking to provide comprehensive care are affected by restrictions on abortion, trainees – and by extension, the broader health system they will staff – are disadvantaged by the inhospitable legal context of Ohio. Whether trainees stay in state without learning necessary skills or depart for practice settings where they can practice in accordance with their professional judgment, abortion restrictions are likely to exacerbate shortages of OB/GYNs in rural and medically underserved communities across vast geographic areas of the U.S. Moreover, those that stay in restrictive states will face ethical dilemmas as their ability to exercise clinical judgment is increasingly constrained[24]. Our findings, and previous research, suggest that this would put patients at risk – especially as healthcare professionals and researchers also anticipate an increase in injuries associated with less safe abortion practices and higher risk pregnancies in the absence of legal options for abortion care. [6–8]

State laws may not be the sole reason for deficient training in abortion care. Our findings suggest that medical schools and residency programs ought to consider their responsibilities in sufficiently preparing their trainees to practice medicine and provide comprehensive reproductive care for their patients in abortion-restrictive states. Moreover, employing institutions must also contemplate their role in ensuring physicians remain competent to provide a full spectrum of OB/GYN care. This might mean providing continuing education opportunities that enable physicians to practice their skillsets or advocating to state legislators about the impact of laws that limit abortion provision in their facilities in order to support both physician and patient well-being.

The landscape of reproductive healthcare in the U.S. is complex, and simply increasing the number of OB/GYNs trained in abortion care is insufficient to redress all its shortcomings. There remains a great need to address abortion stigma, systemic racism, and poverty – all issues that impact reproductive well-being. Still, the need for comprehensive reproductive healthcare will persist, and it would behoove the medical community to be well positioned to meet those needs.

## Limitations

This study has several limitations. Study participants were predominately white women who practiced OB/GYN in metropolitan areas. Additionally, it is possible that physicians who support abortion rights were more likely to participate in the study. Thus, the study topic and participant demographics possibly limited the number of study participants and range of perspectives shared in focus groups and interviews. Future research that includes a more diverse group of participants may reveal additional perspectives on and experiences with Ohio’s abortion laws. Moreover, the voices of current medical students would help to further elucidate the link between Ohio’s abortion laws, what Ohio’s medical students learn about abortion, and what role medical schools might play in expanding educational opportunities about abortion.

## Conclusion

Prior to the reversal of *Roe v Wade* and *Planned Parenthood v Casey*, OB/GYNs in Ohio and other abortion hostile states already experienced barriers to training in abortion care. In the post-*Dobbs* era wherein abortion laws in roughly half of U.S. states severely restrict, completely outlaw, or leave abortion legality in flux as court cases play out, abortion training will become increasingly difficult to access. Based on the previous experiences of OB/GYNs in hostile states, this is likely to leave a large swath of physicians without the skills needed to provide care for their patients – especially in emergent situations. This puts the lives, health, and autonomy of patients at risk and places physicians who wish to care for their patients in precarious ethical positions. Reducing abortion restrictions and expanding protections for abortion access will ensure OB/GYNs have the opportunity to learn and keep skills necessary for the provision of critical reproductive healthcare services; moreover, it will ensure that patients have access to the care they want, need, and deserve.

## Data Availability

In order to protect the privacy and confidentiality of study participants, data collected in this study are not publicly available.

## Appendix 1 Abortion Laws and their Impact on Physicians’ Counseling, Referral, and Clinical Practices: Focus Group and Interview Guide

**Distribute case study of laws**.

- We are going to be discussing the following abortion laws and how they have impacted your ability to counsel and provide reproductive health care to your patients. If time permits, we will also discuss pending and proposed laws and what impact you anticipate they could have on your practices. I will read a brief synopsis of each law before we begin our discussion.
- Before we get started, are there any particular state abortion laws that you would like to discuss today?
  - Note: take notes, prioritize laws currently in effect, signed but not yet in effect, pending litigation, and finally proposed.

**Laws Currently in Effect**:

- **HB 153: Bans public facilities from providing non-therapeutic abortions (2011)**
  - Are you familiar with this law?
    - Probe: what is your reaction to this law?
  - Did the passage of this law impact your ability to provide care for patients? If yes, how so? If no, why not?
    - Probes: abortion provision, provision of other reproductive health care and counseling
  - How did the passage of this law impact your referral practices for your patients who were seeking abortion care? How easy or difficult has it been to find a place to send them?
    - Probes: local/regional/out-of-state referrals
  - After the passage of this law, did any of your patients seeking abortion services state that they were unable to find a place to get an abortion? If so, how did you/your office assist them?

**Laws Currently in Effect**:

- **HB 78: Bans abortion once viability is confirmed and requires viability testing after 20^th^ week. Exceptions for emergencies (2011)**
  - Are you familiar with this bill?
    - What is your reaction to it?
  - What is your understanding of ‘viability testing’ and confirmation of viability?
  - How (if at all) did the passage of this law impact:
    - your ability to provide care for patients?
    - your scheduling practices so as to see pregnant patients earlier in their pregnancies?
    - how you counseled or managed the care of patients seeking abortions?
    - how you counseled patients whose fetuses were diagnosed with anomalies and/or genetic disorders?
    - how you managed the care of patients whose fetuses were diagnosed with anomalies and/or genetic disorders?
    - how you managed referrals and the care of patients in need of medically necessary abortions?

**Laws Currently in Effect**:

- **SB 127**:  **Bans abortion after 20 weeks. Exceptions for emergencies with approval from multiple doctors (2015/2017)**
  - Are you familiar with this law?
    - What is your reaction to this law?
  - Did the passage of this law impact your ability to provide care for patients? If yes, how so? If no, why not?
  - How (if at all) did the passage of this law impact:
    - your patient scheduling practices so as to see pregnant patients earlier in their pregnancies?
    - how you counseled patients whose fetuses were diagnosed with anomalies and/or genetic disorders?
    - how you managed the care of patients whose fetuses were diagnosed with anomalies and/or genetic disorders?
    - how you managed referrals and the care of patients in need of medically necessary abortions?

**Laws Currently in Effect**:

- **HB 294: Prohibits the Ohio Department of Health from distributing funds to agencies that perform or promote nontherapeutic abortions or contract with an organization that performs or promotes nontherapeutic abortions (2016)**

- Are you familiar with this law?
  - Probe: If organizations like Planned Parenthood lose Title X funds and the ability to provide contraceptives, among other services, at a reduced cost, what impact do you think that would have on your practice?

**Legislation signed by the governor, but not currently in effect in Ohio**:

- **HB 214: Would prohibit abortion if reason sought is prenatal Down syndrome diagnosis (2017)**
  - Are you familiar with this law?
    - Probe: What is your reaction to it?
  - Did the passage of this law impact your ability to provide care for patients? If yes, how so? If no, why not?
  - How would the passage of this law:
    - alter the way you counsel patients whose fetuses were diagnosed with Down syndrome?
    - alter the way you counsel patients who were concerned that their fetus may have down syndrome?

- impact your referrals practices for patients whose fetuses are diagnosed with Down syndrome to abortion providers? How difficult would it be to find a place to refer them?

**Legislation signed by the governor, but not currently in effect in Ohio**:

- **HB 145: Would ban dilation and evacuation (D&E) procedure. Exception if the mother’s life is at risk (2018)**
  - Are you familiar with this legislation?
    - Probe: what impact do you foresee this law having on medical practices?
  - Would the enactment of this law impact your ability to provide care for patients? If yes, how so? If no, why not?
  - If this law is enacted, how would you counsel patients who desire or need abortions? Would you have concerns about the impact of such a law on particular types of patients?
    - Probe: patients in their second or third trimesters

- If this law is enacted, how would you manage the care of patients who desire or need abortions? Would you have concerns about the impact of such a law on particular types of patients?

**Legislation signed by the governor, but not currently in effect in Ohio**:

- **SB 23: would ban abortion after detectable heartbeat (2019)**
  - Are you familiar with this proposed bill?
    - Probe: what impact do you foresee this bill having on medical practices?
    - Probe: how do you keep track of legislation – when it passes, fails, or is reintroduced? How do you interpret the reintroduction of bills like the ‘6-week’ ban?

- If it were to go into effect, how would:
  - this law impact your ability to provide care for patients?
  - you counsel patients who desire or need abortions? Would you have concerns about the impact of such a law on particular types of patients?
    - Probe: patients who discover they are pregnant after 6 weeks, unviable pregnancies, fetal anomalies or genetic disorders, or patients seeking medically necessary abortions?
  - you manage the care of patients who desire or need abortions? Would you have concerns about the impact of such a law on particular types of patients?
    - Probe: patients who discover they are pregnant after 6 weeks, unviable pregnancies, fetal anomalies or genetic disorders, or patients seeking medically necessary abortions?

**Proposed Legislation**

- **HB 565 (Proposed 2018): Would ban abortion outright with no exceptions.**
  - Are you familiar with this proposed bill?
    - Probe: what impact do you foresee this bill having on medical practices?
  - Would the passage of this law impact your ability to provide care for patients? If yes, how so? If no, why not?
  - If this law passed, how would you counsel patients who desire or need abortions? Would you have concerns about the impact of such a law on particular types of patients?
    - Probe: patients with unviable pregnancies, fetal anomalies or genetic disorders, or patients seeking medically necessary abortions?
  - If this law passed, how would you manage the care of patients who desire or need abortions? Would you have concerns about the impact of such a law on particular types of patients?
    - Probe: patients with unviable pregnancies, fetal anomalies or genetic disorders, or patients seeking medically necessary abortions?
